# Effects of yacon flour associated with an energy restricted diet on intestinal permeability, fecal short chain fatty acids, oxidative stress and inflammation markers levels in adults with obesity or overweight: a randomized, double blind, placebo controlled clinical trial

**DOI:** 10.20945/2359-3997000000225

**Published:** 2020-03-30

**Authors:** Adriane Moreira Machado, Nayara Benedito Martins da Silva, Renata Maria Pereira de Freitas, Mariella Bontempo Duca de Freitas, José Benício Paes Chaves, Leandro Licursi Oliveira, Hércia Stampini Duarte Martino, Rita de Cássia Gonçalves Alfenas

**Affiliations:** 1 Universidade Federal de Viçosa Departamento de Nutrição e Saúde Viçosa MG Brasil Universidade Federal de Viçosa, Departamento de Nutrição e Saúde, Viçosa, MG, Brasil; 2 Universidade Federal de Viçosa Departamento de Tecnologia de Alimentos Viçosa MG Brasil Universidade Federal de Viçosa, Departamento de Tecnologia de Alimentos, Viçosa, MG, Brasil; 3 Universidade Federal de Viçosa Departamento de Biologia Animal Viçosa MG Brasil Universidade Federal de Viçosa, Departamento de Biologia Animal, Viçosa, MG, Brasil; 4 Universidade Federal de Viçosa Departamento de Biologia Geral Viçosa MG Brasil Universidade Federal de Viçosa, Departamento de Biologia Geral, Viçosa, MG, Brasil

**Keywords:** Yacon, white blood cells, obesity, intestinal permeability, oxidative stress

## Abstract

**Objectives:**

Yacon flour is rich in bioactive compounds (phenolic compounds and fructooligosaccharides (FOS)), and may therefore reduce the risk of diseases associated with excess body weight. However, its effect on fecal short chain fatty acids (SCFA), intestinal permeability, oxidative stress and inflammation markers has not been studied in adult humans with excess body weight. Thus, we evaluated the effect of the consumption of yacon flour on these variables.

**Materials and methods:**

Twenty-six excess body weight (30.4 ± 2.4 kg/m^2^) adults (31.3 ± 8.5y) were randomized to one of two groups (yacon flour or control; n = 13) on a double blind clinical trial. Subjects received a breakfast drink containing or not yacon flour (25g) associated with an energy restricted diet, for six weeks. The flour chemical characterization, FOS and total phenolics contents were evaluated. Antioxidant capacity was evaluated in vitro and in vivo (plasma). Intestinal permeability, fecal SCFA, oxidative stress and inflammatory markers also were evaluated in vivo.

**Results:**

Yacon flour was well tolerated. It presented an in vitro and in vivo antioxidant capacity, increased plasma total antioxidant capacity (Δ_YAC_: 49.16 (-4.20; 156.63)) and reduced protein carbonyl concentrations (Δ_YAC_: -0.98 (-1.54; -0.42)). A reduction in SCFAs was observed in both groups (Δ_acetic_: -3.16 (-5.07; -0.95) vs. -1.05 (-2.65; 1.11); Δ_propionic_: -1.05 (-2.60;-0.38) vs. -0.41 (-2.08; 0.09); Δ_butyric_: -0.75 (-1.38; -0.04) vs. -0.28 (-0.98; 0.11), for YAC and CON, respectively). Other variables did not change.

**Conclusion:**

The yacon flour increased the plasma antioxidant capacity, decreased oxidative stress and SCFAs in adults with obesity or overweight.

## INTRODUCTION

Yacon root and its products seem to control inflammation ( 1 ) and reducing body weight and body fat mass ( 2 ) in humans, besides improving oxidative stress ( 3 , 4 ), in studies *in vitro* and in animal models, thus reducing the risks of cardiovascular disease, type 2 diabetes and other chronic diseases. Recent studies identified the existence of an association between yacon consumption and intestinal barrier function improvement ( 5 ). These effects seem to occur due to its high fructooligosaccharides (FOS) and phenolic compounds content, mainly chlorogenic acid, present in yacon ( 6 , 7 ).

Fructooligosaccharides are prebiotics fermented by intestinal bacteria, leading to the production of lactic acid, short chain fatty acids (SCFAs), mainly acetic, propionic and butyric acid, and gases. Studies have suggested that FOS and its fermentation products may affect the composition of the microbiota ( *Lactobacillus* and *Bifidobacterium* spp), promoting a healthier gut microvillus environment ( 6 , 8 ). Prebiotic consumption may also increase the expression of tight junctions from the intestinal epithelium. The increase in these proteins was correlated with reduced intestinal permeability, and reduced plasma and hepatic tissue proinflammatory cytokines and oxidative stress markers expression ( 9 ). The phenolic compounds in turn contain hydroxyl groups capable of donating electrons and thus reduce free radicals ( 10 ). However, due to the *in natura* root high water content, FOS is hydrolyzed and phenolic compounds are inactivated in response to polyphenols oxidases action. On the other hand, FOS and phenolic compounds are stable in dehydrated products, such as yacon flour ( 7 , 11 ).

Few studies have evaluated the functional effects of yacon flour in humans ( 2 , 12 , 13 ). To the best of our knowledge, this is the first clinical trial to investigate the effect of yacon flour consumption on faecal SCFAs concentrations, intestinal permeability, oxidative stress and inflammatory markers concentrations in excess body weight adults. The aim of this study was to evaluate the effect of the consumption of a drink containing yacon flour associated with an energy restricted diet on such variables in adults with excess body weight.

## MATERIALS AND METHODS

### Yacon flour composition

Yacon flour was purchased from Linea Verde Alimentos (Curitiba, Brazil) and was stored at 21°C and 64% humidity until the moment of use. Yacon flour chemical composition and FOS content was determined as previously described ( 2 ).

To determine the total phenolic content, a yacon flour extract was prepared. To obtain the extract, 12 g of yacon flour were homogenized with 120 mL of 50% methanol. That mixture stayed for 1 hour at room temperature. Subsequently, the sample was centrifuged (7,000 xg, 15 min), the supernatant collected and kept under refrigeration (4°C). The residue was submitted to a second extraction with 120 mL of 70% acetone at room temperature for 1 hour. After that time, the sample was centrifuged once more (7,000 xg. 15 min) and the supernatant was transferred to a volumetric flask containing the first supernatant and the volume (200 mL) was completed with distilled water. Before the analysis, the extract was filtered on filter paper, Whatman #1, in vacuum pressure, and concentrated in a rotary evaporator (MA 120, Marconi, São Paulo, Brazil) at 40°C, the concentrate was resuspended in distilled water to obtain a final volume of 50 mL.

Phenolic compounds content determination was carried out according to the methodology described by Singleton and Rossi ( 14 ) with some adaptations. In an assay tube an aliquot (0.6 mL) of the extract obtained (diluted 1/25) and 3 mL of the Folin-Ciocauteau reagent (diluted 1/10) were added and the mixture was stirred. After three minutes 2.4 mL of saturated sodium carbonate (7.5% m/v) were added. The mixture was allowed to rest in the dark for 1 hour and a UV-Visible (BEL Engineering UV-M51) spectrophotometer was read at 760 nm. Gallic acid was used as standard and the results were expressed in milligrams of gallic acid equivalents (GAE) per 100 grams of yacon flour.

### Antioxidant potential in vitro

Yacon flour antioxidant capacity was assessed using the 2,2-azinobis-3-ethylbenzothiazoline-6-sulfonic acid (ABTS•+) ( 15 ) and 2,2-Diphenyl-1-picrylhydrazyl (DPPH) ( 16 ) assays with some modifications (for details, see Supplementary material). The results were expressed as Trolox equivalent antioxidant capacity (TEAC) (μmol TEAC. g-1 of flour). All analyses were performed in duplicate.

### In vivo effects

#### Study design

This is a double-blind, parallel, randomized, placebo-controlled, two-arm, clinical trial study involving adults with excess body weight. The intervention lasted six weeks (± 5 days). A tolerance of ± 5 days was adopted to ensure that the assessments were not made in the menstrual period in women. Potential subjects were screened for eligibility and randomly allocated to control group (1:1) (CON, n = 13) or yacon flour group (YAC, n = 13), using the block randomization technique (with block size equal to 4). This technique was applied by an independent research group not involved in the study.

During the study, on week days subjects daily attended the Laboratory of Food Intake, Department of Nutrition and Health, Federal University of Viçosa – Brazil, to consume a breakfast drink (350 mL) without yacon flour (control) or with 25g of yacon flour ( Table 1 ), according to the group in which they were allocated, as part of the energy-restricted diet (-500 kcal/day) individually prescribed to each subject. On weekends, identical breakfasts were provided to be consumed at home. Adherence to the protocol on weekends was verified by asking the subjects about the consumption of the breakfast provided. In addition, subjects were asked to bring to the laboratory any quantity of food supplied but not ingested on weekends. A trained investigator, unrelated to data collection and analyses, was responsible for assessing adherence to the protocol, preparing and serving the drinks. To avoid monotony and increase adherence to the study protocol, seven different flavors of drinks (cappuccino, cocoa milk, coffee with milk, guava, mango, blackberry and passion fruit vitamins), with similar nutritional composition, were served.

**Table 1:** Nutritional composition1 of the seven rotating breakfast drinks served

	Energy (kcal)		Carbohydrate (g)		Protein (g)		Fat (g)		Fiber (g)^2^	

	CON	YAC	CON	YAC	CON	YAC	CON	YAC	CON	YAC
Guava vitamin	441.4	462.6	65.67	68.43	15.08	15.78	13.16	13.98	11.36	22.55
Blackberry vitamin	440.7	461.9	63.92	66.68	16.66	17.36	13.16	13.98	0.51	11.70
Passion vitamin	430.9	452.18	63.92	66.68	14.21	14.91	13.16	13.98	1.91	13.1
Mango vitamin	426.7	447.9	63.92	66.68	13.16	13.86	13.16	13.98	2.43	13.62
Cappuccino	394.7	412.9	55.44	58.80	13.41	14.11	13.26	13.48	0.75	11.7
Cocoa milk	394.74	412.9	55.44	58.80	13.41	14.11	13.26	13.48	0.75	11.7
Coffe with milk	397.43	411.6	53.55	55.90	13.16	13.86	14.51	14.73	0.75	11.7
Mean	418 ± 21.6	437 ± 23.9	60.2 ± 5.2	63.1 ± 5.1	14.1 ± 1.3	14.8 ± 1.3	13.4 ± 0.5	13.9 ± 0.4	2.6 ± 3.9	13.7 ± 3.9

^1^ Based on the information contained in the food labels and on yacon flour nutritional composition analysis. ^2^ Dietary fiber: total fiber (soluble + insoluble) + fructooligosaccharides. CON: control group; YAC: yacon flour group.

Diets were prescribed considering the nutritional composition of the estimated energy requirement, the level of physical activity and the breakfast shakes daily consumed in the laboratory during the study. The diets prescribed to had CON and YAC groups had similar calories and macronutrients contents (1,734 ± 494.2 kcal *vs.* 1,729 ± 471.1 kcal, carbohydrate: 51.6 ± 3.6% E *vs.* 51.3 ± 2.4% E, protein: 21.2 ± 2.4% E *vs.* 21.0 ± 1.9% E, fat: 28.8 ± 2.4% E *vs.* 29.1 ± 2.0% E, for CON and YAC, respectively) ( 2 ). Adherence to the prescribed diet was monitored using 3-day (2 weekdays and 1 weekend) food records applied in the third and in the last week of the study. Subjects were instructed to maintain a constant level of physical activity throughout the study. If subjects presented infection and/or inflammation symptoms, or, if women had menstrual changes, the subject’s permanence in the study was reevaluated. However, no volunteer was excluded because of these changes. The results of food intake, body composition, anthropometric, intestinal function and biochemical variables were previously published ( 2 ).

#### Subjects

Four hundred and four (404) subjects were initially recruited through local advertisements flyers, radio, newspaper and electronic media, from August 2017 to May 2018. Eligible subjects were adult men and women (20-45 years), with excess body weight (BMI 25-35 kg/m^2^), who consumed regular breakfast, had low physical activity level (according to the International Physical Activity Questionnaire) and dietary restraint ≤ 14 ( 17 ). The exclusion criteria were: smokers, pregnant/lactating, and people with: habitual consumption of more than 30 g of alcohol/day; use of medications that affect glycemia or energy metabolism; use of medications, herbs or diets to reduce appetite and body weight; body weight gain or loss > 5 kg three months prior to the beginning of the study; recent change in the level of physical activity; aversion or intolerance to the food provided in the study; existence or history of endocrine, cardiovascular, arterial hypertension, liver and/or gastrointestinal diseases; report of eating disorders; use of laxatives or antibiotics three months prior to the beginning of the study; use of probiotics, prebiotics or symbiotics (> 2/week) in the month prior to the beginning of the study, and women with menstrual irregularity (three months prior to the beginning of the study).

**Figure 1:**
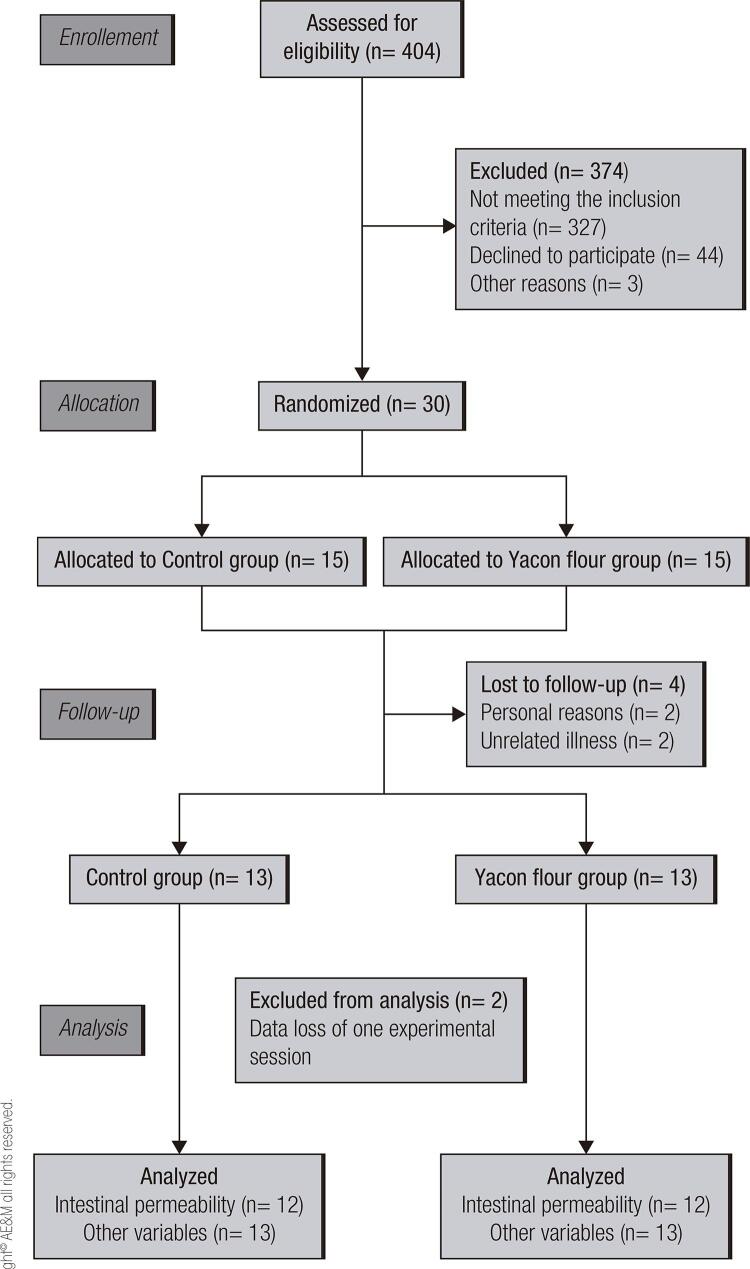
CONSORT study flow diagram.

From the 404 recruited subjects, 30 filled out the criteria for inclusion and were allocated in one of the study groups. Of these, four subjects did not complete the study protocol. Besides, intestinal permeability analyses from two subjects were lost ( Figure 1 ). No exclusions happened due to protocol non-compliance.

The study was conducted according to the Declaration of Helsinki and is registered in the Brazilian Clinical Trials Registry (number RBR-6YH6BQ). All procedures involving human subjects were approved by local ethical committee (Universidade Federal de Viçosa, number 62047316.6.0000.5153). Written informed consent was obtained from all subjects.

#### Faecal short chain fatty acids (SCFAs) concentrations

Faecal samples were collected, before and after the intervention period, into empty sterile flasks and immediately frozen and kept at -80°C, pending analysis. Faecal samples (500 mg) were homogenized in 1 mL of Milli-Q water and centrifuged (12,000 xg, 10 min) and the cell-free supernatants were treated as described by Siegfried and cols. ( 18 ).

The concentration of SCFAs was determined by high performance liquid chromatography (HPLC). The samples were analyzed using a chromatograph Dionex Ultimate 3000 Dual coupled to a Shodex RI-101 refractive index detector maintained at 40°C, and Phenomenex ion exchange column Rezex ROA, 300 x 7.8 mm maintained at 40°C. The mobile phase used was composed of 5mM sulfuric acid (H2SO4) with a flow rate of 0.7 mL/min. The organic acids used for calibration of the standard curve were: acetic, propionic, butyric and crotonic acids.

#### Intestinal permeability

**Supplementary figure 1:**
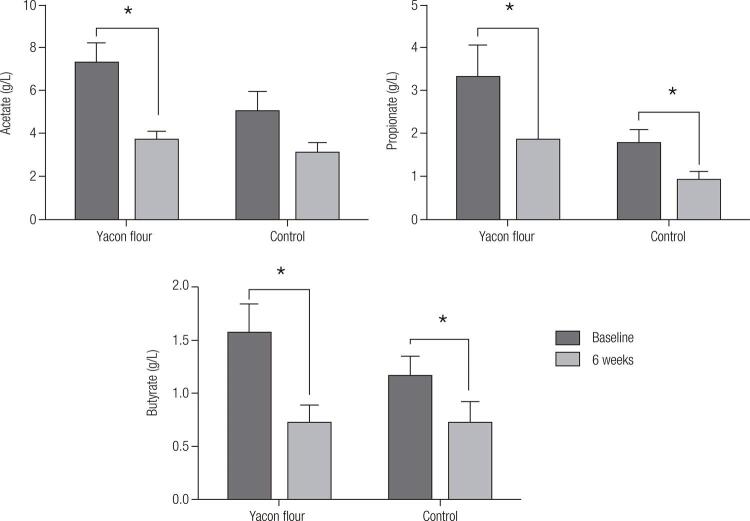
Data collection protocol.

The subjects were instructed not to consume dietary sources of the lactulose and mannitol ( 19 ) two days before the assessment. After 12 hours of fasting, the subjects attended the laboratory and were instructed to eliminate any residual urine. An iso-osmolar solution, containing 6.67 g of lactulose and 2.0 g of mannitol diluted in water to complete 150 mL of solution, was ingested by the subjects. The osmolarity of the solution is similar to that of plasma, in order to avoid any damage to the studied epithelium. Breakfast, containing or not containing yacon flour, was offered 2 hours after the solution was ingested. 800 mL of water were given at regular intervals to the subjects. During six hours, all voiding product was collected (for details, see Supplementary material, Figure 1). Next, 15 mL of urine was transferred to a single vial containing 12 mg of thimerosal to prevent bacterial growth. The samples were stored in a freezer at -80°C until analysis.

Two milliliters of stored urine samples were taken into a water bath (56°C, 10 min), centrifuged (10,000 rpm, 7 min) and filtered through a microporous membrane (0.22 μm x 13 mm, Millipore, USA). Urinary lactulose and mannitol excretions were analyzed by HPLC, Dionex Ultimate 3000 Dual coupled to a Shodex RI-101 refractive index detector maintained at 40°C, and Phenomenex Rezex ROA ion exchange column, 300 x 7.8 mm maintained at 40°C. The volume of filtered urine injected was 20 μL. The mobile phase used was 5 mM of sulfuric acid with a flow rate of 0.7 mL/min. Standardization curves with lactulose and mannitol standards were used to determine sugar concentration in the urine samples. The net amount of sugar excreted was calculated by multiplying the determined concentration of each sugar in the urine by the total volume of urine collected over the course of 6 hours. Then the sugar dose administered was used to calculate the percentage of lactulose (%L) and mannitol (%M) excreted in the urine. These results were used to calculate the proportion of lactulose/mannitol (L/M).

#### Metabolic biomarkers

One week before the beginning of the study, subjects were instructed not to consume alcoholic drinks and not to change their usual food intake. A standard dinner (200 mL of Tial^®^ nectar (grape flavor), 85 g of pasta and 10 g of Parmesan cheese -523 kcal, 57.4% CHO, 10.3% PTN, 32.3% LIP, 2.1g fibers) was supplied to be consumed the night before the evaluations.

Antecubital blood samples were collected after 12 hours of fasting. Immediately after collection, blood samples were sent to the laboratory to complete blood count (leukocytes, lymphocytes, neutrophils and platelets). The remaining of the blood was centrifuged for serum and plasma separation (3500 rpm, 4°C, 15min), and immediately frozen at -80°C until analyses.

C-reactive protein (CRP) was assessed by the quantitative method based on immunoturbidimetric, using commercially available kit for ultrasensitive CRP (Bioclin^®^ MG, Brazil). The neutrophils/lymphocytes (NLR) and the platelets/lymphocytes (PLR) ratios were calculated.

#### Oxidative stress

Catalase activity (CAT) ( 20 ), glutathione S-transferase (GST) ( 21 ), malondialdehyde (MDA) ( 22 ), protein carbonyl ( 23 ), nitric oxide (NO) ( 24 ) and antioxidant capacity (by the ferric reducing antioxidant power (FRAP) assay) ( 25 ) were evaluated in plasma.

## Statistical analysis

The present study presented a statistical power of 99.9 % (α = 0.05) to detect a reduction of 15% in the FRAP concentration and a power of 86.7% (α = 0.05) to detect a reduction of 4.5 mg/L in the CRP concentration, considering our subjects *baseline* data ( 26 ). Antioxidant capacity was therefore considered as the primary outcome, whilst the others oxidative stress and inflammatory markers, intestinal SCFAs concentrations and intestinal permeability were measured as secondary outcomes.

Statistical analyses were conducted using SPSS software (SPSS Inc., Chicago, IL, 2015, version 22.0). Data normality and homoscedasticity were evaluated by the Shapiro-Wilk and Levene tests, respectively. The effect of the intervention was assessed by comparing the outcome variables intra and inter CON and YAC groups using generalized estimating equation model (GEE) adjusted by sex. We used Bonferroni post-hoc to identify the differences on group, time and group*time interaction when required. For the variables with normal distribution, a connection identify function was used. For the variables that did not follow normal distribution, gamma distribution with log link was used. The working correlation matrix used was unstructured and robust estimator covariance matrix. These models were adjusted by baseline values. Α α < 0.05 was adopted as the level of statistical significance.

## RESULTS

### Yacon flour chemical characterization and in vitro antioxidant capacity

The portion of yacon flour tested in the present study (25 g) contained 0.69 g of protein, 0.21 g of fat, 1.23 g of ashes and 21.36 g of total carbohydrate. It contained 11.72 g of dietary fiber, of which 8.67 g were FOS. Total phenolic compounds content was 164 mg of GAE.

Yacon flour antioxidant capacity was 54.2 μmol TEAC. g^-1^ of flour by the DPPH method and 22.18 μmol TEAC. g^-1^ of flour by the ABTS method.

### In vivo outcomes

The groups were homogeneous at the beginning of the study. Subjects were mostly female (57.7%) and obese (57.7% *vs.* 42.3% overweight according to BMI). None of the subjects had systolic blood pressure greater than 139 mmHg or diastolic blood pressure greater than 90 mmHg and glycemia greater than 5.5 mmol/L (data not shown). Two subjects from YAC group and one from CON group had concentrations of CRP above 10 mg/L, suggesting the presence of acute inflammation or infection at baseline. However, the removal of these subjects from the analyses did not alter the results of the comparisons. Therefore, the subjects were kept in their respective groups. During the first days of intervention, approximately 15% of the YAC group subjects reported abdominal discomfort symptoms, such as flatulence and abdominal pain. These effects decreased after the second week and no subjects were excluded from the study due to protocol non-compliance or drinks non-acceptance.

**Figure 2:**
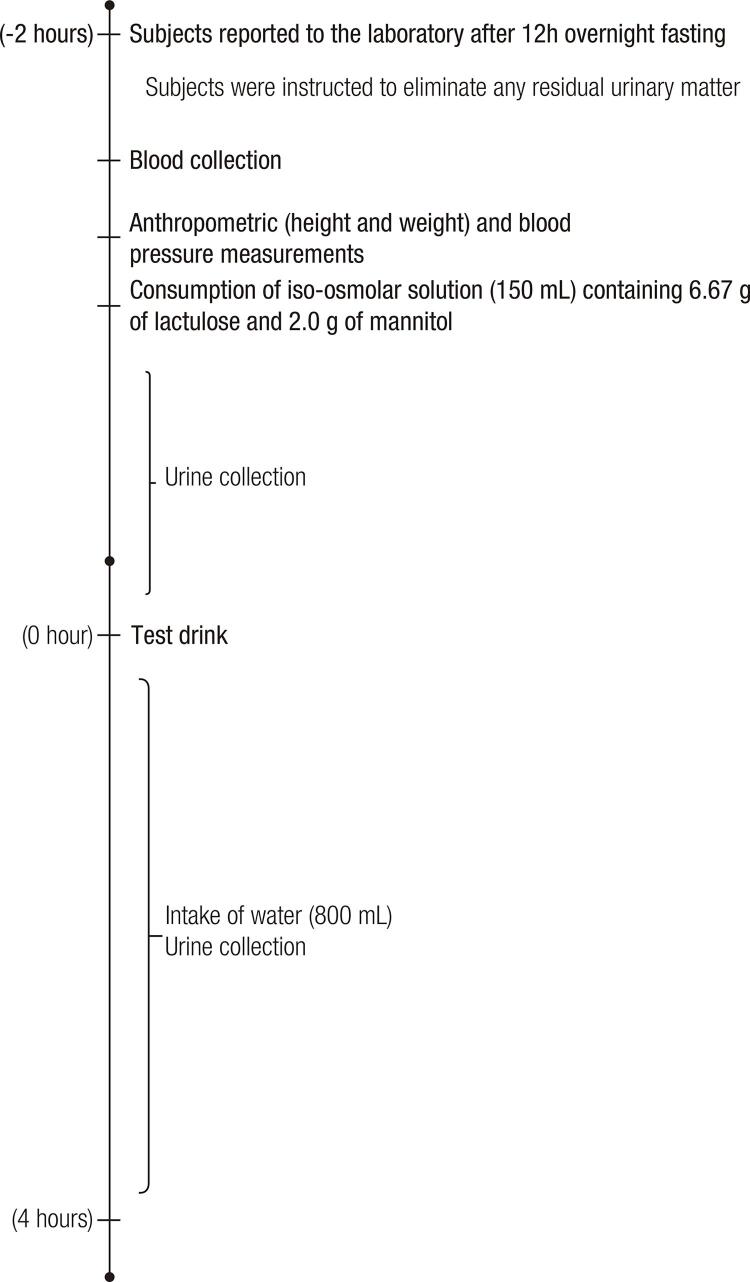
Mean ± SEM changes (Δ values = 6 weeks values – baseline values) in short chain fatty acids (acetate, propionate, butyrate) in response to consumption of a drink not containing (control, n = 13) or containing yacon flour (25g, n = 13) allied to energy-restricted diet (-500 kcal/d). Results were obtained analyzing faecal samples. *Significant intra-group reduction (generalized estimating equation model (GEE), p < 0.05).

The concentrations of propionic and butyric acid significantly reduced in both groups after six weeks of intervention. Besides, acetic acid reduced only in the YAC group ( Figure 2 ).

No statistical differences were detected in the urinary lactulose or mannitol excretion, the lactulose/mannitol ratio, CRP, NLR and PLR concentrations, platelet count and white blood cell counts between baseline groups or after six weeks of intervention ( Tables 2 and 3 ). Of the YAC group subjects that had NLR increased at baseline, in 50% of them, the values reached normality. On the other hand, that reduction was verified in 20% of the CON group subjects.

**Table 2:** Intestinal permeability assessments* at baseline and after intervention (6 weeks) according to experimental groups**

	Control group (n = 12)		Yacon flour group (n = 12)		*P*_inter_

	Baseline	6 weeks	Baseline	6 weeks	

	Adjusted mean ± SEM	Adjusted mean ± SEM	Adjusted mean ± SEM	Adjusted mean ± SEM	
% Lactulose excretion	0.92 ± 0.43	2.92 ± 0.29	1.34 ± 0.44	1.79 ± 0.35	0.429
% Mannitol excretion	4.61 ± 1.56	3.95 ± 0.85	5.54 ± 1.92	3.13 ± 0.90	0.484
L/M ratio	0.32 ± 0.08	0.64 ± 0.06	0.40 ± 0.16	0.66 ± 0.13	0.698

^* ^Results obtained by 6-hour urine collection.

^** ^Data are given as adjusted mean and SEM per treatment group. No significant difference occurred intra-group or between-groups (by generalized estimating equation model (GEE), p > 0.05). P inter: between-group comparisons at 6 weeks was adjusted for baseline.

**Table 3:** Inflammatory markers assessments* at baseline and after intervention (6 weeks) according to experimental groups**

	Control (n = 13)		Yacon flour (n = 13)		*P*_inter_

	Baseline	6 weeks	Baseline	6 weeks	

	Adjusted mean ± SEM	Adjusted mean ± SEM	Adjusted mean ± SEM	Adjusted mean ± SEM	
Leukocytes (mm^3^)	6338.58 ± 474.68	6266.96 ± 538.22	6952.60 ± 334.45	6788.12 ± 423.70	0.804
Neutrophils (mm^3^)	3657.07 ± 363.34	3773.76 ± 466.87	3951.24 ± 351.18	3879.20 ± 317.55	0.560
Lymphocytes (mm^3^)	2355.27 ± 208.02	2094.36 ± 157.24	2448.14 ± 248.51	2424.11 ± 213.34	0.206
Platelet (10^3^/mm^3^)	206.06 ± 131.85	198.58 ± 134.17	208.90 ± 170.23	216.19 ± 164.83	0.160
Neutrophil/lymphocyte rate	1.61 ± 0.21	1.81 ± 0.19	1.76 ± 0.24	1.63 ± 0.20	0.100
Platelet/Lymphocyte rate	91.20 ± 7.84	97.86 ± 8.22	91.02 ± 11.45	92.23 ± 11.56	0.443
C reactive protein	2.52 ± 0.84	2.58 ± 0.86	4.89 ± 1.07	5.23 ± 1.17	0.623

* Results were obtained analyzing fasting blood (complete blood count (leukocytes, lymphocytes, neutrophils and platelets) and plasma (CRP)) samples.

** Data are given as adjusted mean and SEM per treatment group. No significant difference occurred intra-group or between-groups (by generalized estimating equation model, p > 0.05). P inter: between-group comparisons at 6 weeks was adjusted for baseline.

The antioxidant enzymes CAT and GST activities, the oxidation product malondialdehyde and nitric oxide concentrations were not affected during the study. At the end of the experimental period, YAC group present an increase in the plasma antioxidant capacity (FRAP method) and reduction in the concentrations of protein carbonyl ( Table 4 ).

**Table 4:** Oxidative stress markers assessments* at baseline and after intervention (6 weeks) according to experimental groups**

	Control (n = 13)		Yacon flour (n = 13)		*P*_inter_

	Baseline	6 weeks	Baseline	6 weeks	

	Adjusted mean ± SEM	Adjusted mean ± SEM	Adjusted mean ± SEM	Adjusted mean ± SEM	
Catalase (U/mg)	1359.09 ± 8.99	1532.11 ± 134.11	1444.3 ± 121.5	1698.64 ± 129.67	0.661
GST (µmol/min/g)	1.50 ± 0.21	1.54 ± 0.18	1.38 ± 0.17	1.44 ± 0.20	0.642
FRAP (µmol/L)	708.75 ± 54.60	727.10 ± 63.21	755.20 ± 50.69^a^	834.38 ± 47.37^b^	0.235
MDA (µmol/mL)	7.42 ± 1.34	0.76 ± 0.98	7.15 ± 1.14	8.36 ± 1.00	0.247
Nitric oxide (µmol/mg)	2.13 ± 0.80	1.51 ± 0.43	1.29 ± 0.29	1.74 ± 0.34	0.054
Protein carbonyl (nmol/mL)	3.05 ± 0.45	2.44 ± 0.34	3.03 ± 0.36^a^	1.94 ± 0.33^b^	0.315

FRAP: ferric reducing antioxidant power; GST: glutathione S-transferase; MDA: malondialdehyde.

^*^ Results were obtained analyzing fasting blood samples (plasma).

^** ^Data are given as adjusted mean and SEM per treatment group. Means followed by different lowercase letters, in the same line, indicate intra-group difference by generalized estimating equation model, p < 0.05. P inter: between-group comparisons at 6 weeks was adjusted for baseline.

## DISCUSSION

To the best of our knowledge, this study provides the first clinical evidence that six weeks of yacon flour consumption reduces oxidative stress status in adults with excess body weight.

Yacon is a source of phenolic compounds (658 mg GAE/100g of flour), mainly chlorogenic acid, that could neutralize free radicals and improve oxidative stress ( 3 , 4 , 7 , 27 ). These compounds contain hydroxyl groups, which can donate electrons and thus reduce free radicals’ production, avoiding biomolecules oxidation ( 10 ). Several methods have been developed to determine food antioxidant potential. However, to measure the antioxidant activity of food extracts, at least two test systems have been recommended ( 28 ). For this reason, the antioxidant activity of the yacon flour was measured using the ABTS and DPPH methods. The total antioxidant activity of the yacon flour was 54.2 μmol Trolox/g of flour by the DPPH method and 22.18 μmol Trolox/g of flour by the ABTS method. It is well known that the antioxidant capacity *in vitro* of food do not necessarily represent its antioxidant potential *in vivo* ( 3 ). Therefore, we used several biochemical assays to determine the *in vivo* antioxidant properties of yacon flour.

In our study, after six weeks of yacon flour consumption, the plasma FRAP of the YAC group was significantly increased while the protein carbonyl level was significantly decreased than the baseline value, in contrast to the CON group, which no differences were observed. Similar results were observed in animal models. Yacon extract supplementation (0.34g of FOS/kg body weight/day, for 14 or 90 days) reduced the concentrations of oxidative stress markers in diabetic rats ( 3 , 4 ). In hypercholesterolemic animals, similar results were observed. Oliveira and cols. ( 27 ) provided yacon root or leaf (20 and 40 mg/kg body weight/day) extracts for 14 days and observed reductions in the concentrations of oxidation products (malondialdehyde and protein carbonyl) in plasma and increase in antioxidant defense (catalase, superoxide dismutase and glutathione peroxidase) in erythrocytes.

An increase in plasma antioxidant capacity provides greater protection against free radicals. There is also a reduction in protein carbonyl concentration, which helps reduce key events in the development of cardiovascular disease ( 29 ). The fact that the plasma FRAP increased after the consumption of yacon flour and no change has been observed in antioxidant enzymes suggests that the substances presents in yacon flour may increase antioxidant capacity without requiring change in the antioxidant defense system.

We observed lower butyrate and propionate after six weeks of YAC and CON breakfast drink associated with an energy restricted diet. Weight-loss per si and weight loss interventions have been shown to induce decreases in fecal SCFA ( 30 ). Since we have previously demonstrated reduced body weight after six weeks intervention ( 2 ), we believe that the decreases in SCFA concentrations may be an indication of reduced efficiency with which energy is harvested from dietary SCFAs during weight loss among overweight or obese individuals ( 31 ). In agree with this, lower fecal concentrations of SCFAs have been associated with the lean phenotype in human studies ( 32 ). Besides, greater mucosal absorption and utilization of SCFAs in peripheral tissues and colonocytes in response to caloric restriction has been proposed to explain lower concentrations fecal SCFA after weight loss ( 30 ). Thus, the amounts of SCFAs quantified in feces could represent a balance between the amounts produced in the large intestine and that which is absorbed or utilized ( 33 ). A weight loss may therefore be associated with increased absorption and utilization of SCFAs, and thus lower concentrations quantified in feces.

Yacon flour consumption did not affect intestinal permeability. To our knowledge, the effect of yacon consumption on human intestinal permeability hasn’t been assessed by any other author. In animals with colon cancer, yacon flour consumption (7.5% FOS, during 8 weeks) reduced urinary mannitol and lactulose excretion ( 5 ). It should be considered, however, that in the present study yacon flour was consumed by adults with excess body weight and that did not have a significant intestinal integrity impairment, such as in colon cancer. In addition, differences in FOS concentration and in the intervention, duration may have contributed to the differences in the outcomes.

Daily consumption of yacon flour associated with an energy restricted diet also did not affect the concentration of most of the inflammatory variables evaluated in this study. However, 67% of subjects who consumed yacon flour showed a statistically non-significant reduction in NLR at the end of the study compared to baseline, and in half of them the values were below the proposed cut-off point (1.84) ( 34 ). Buyukkaya and cols. ( 34 ) observed that subjects with NLR > 1.84 had higher glucose concentration and plasma C-reactive protein and greater number of criteria for metabolic syndrome. Thus, a reduction of NLR as observed in our study may indicate a beneficial effect of yacon in reducing the inflammatory status and improving general health status.

It should be emphasized that the subjects in this study did not present metabolic complications, as in studies in which there was a reduction on inflammatory markers after yacon supplementation ( 1 , 27 ). Thus, it is possible that yacon flour exerts significant effects on inflammatory markers only in metabolically decompensated individuals. Results from healthy animal studies receiving standard diet supplemented with yacon corroborate our hypothesis. Supplementation with yacon flour (0.34 and 6.8g of FOS/kg body weight/day) did not affect leukocyte, lymphocyte, platelet or neutrophil counts in healthy animals ( 8 , 35 ).

To our knowledge, this is the first study evaluating the effects of yacon flour consumption associated with an energy restricted diet on intestinal permeability, oxidative stress, and inflammation markers in adults with excess body weight. Yacon flour was chosen as our test food because its FOS and phenolic compounds contents are more stable than the ones in yacon root. Our data were obtained in a double-blind manner and were double-typed. In addition, we used strict eligibility criteria to select our subjects and the consumption of the test food (yacon flour) was done in the laboratory to guarantee adherence to the protocol. Our intervention did not produce adverse effects. Subjects presented a good adhesion and acceptability to our protocol. However, the limitations of our study were that we did not assess the intestinal microbiota and the fact that SCFAs concentrations were assessed in the feces. However, it is difficult to precisely access SCFAs production because the value obtained in the feces is a balance between its production and absorption. In addition, evaluating fecal SCFAs concentration has been one of the most widely method used by other researchers.

In conclusion, the consumption of 25g of yacon flour associated with an energy restricted diet for 6 weeks by adults with excess body weight increased the total antioxidant capacity and decreased the oxidative stress and SCFAs and, did not affect the concentration of most of the inflammatory variables. Whether the beneficial effects of this short-term yacon flour supplementation will maintain in the longer term is not clear. Assessing the efficacy of yacon flour in the long-term period is therefore warranted. New studies should be conducted to verify that.

### Supplementary material

#### *Antioxidant potential* in vitro

DPPH ^•^ (2,2-Diphenyl-1-picrylhydrazyl) assay was performed according to the methodology proposed by Brand-Williams and cols. ( 16 ). A total of 0.5 mL of the extract was mixed with 3.5 mL of the DPPH• solution (60 μmol L^-1^). The samples were kept for one hour at room temperature in a dark environment. The absorbance was then read at 517 nm in a UV-VISIBLE spectrophotometer (BEL Engineering UV-M51). The results were expressed as Trolox equivalent antioxidant capacity (TEAC) (μmol TEAC g^-1^ of flour).

For ABTS^•+^ (2,2’-azinobis-3-ethyl-benzothiazoline-6-sultonated) radical formation, a methodology described by Re and cols. ( 15 ) was used with some modifications. ABTS^•+^ radical was formed from the addition of ABTS aqueous solution (7 mmol L^-1^) to a potassium persulfate solution (2.45 mmol L^-1^) in a ratio of 1:1. The mixture was heated in a water bath (40°C, 30 min), and the solution absorbance was corrected to 0.70 (± 0.05) at 734 nm with the addition of 80% ethanol. An aliquot of 0.5 mL of the extract, in different concentrations, was transferred to the test tubes with 3.5 mL of the ABTS^•+^ radical. The reaction occurred in the dark (6 min) and the absorbance was read at 734 nm in UV-Visible Spectrophotometer (UV-M51, BEL Engineering, Monza, Italy). The results were expressed as TEAC (μmol TEAC. g^-1^ of flour). All the analyses were performed in duplicate.

## Oxidative stress

Catalase activity was measured according to Hadwan and Abed ( 20 ) with modifications. An aliquot of plasma (5 µL) was added to 100 µL substrate (65 mmol/mL H2O2 in 60 mmol/l sodium potassium phosphate buffer, pH 7,4) or in 100 µL buffer (white) and incubated at 37ºC. After three minutes. the reaction was stopped with the addition of 150 µL molybdate and the absorbance reading was taken at 374 nm. Changes in absorbance, in relation to white, were recorded. The absorbance conversion into micromolar concentrations of H2O2 was calculated from a standard curve using a known concentration of H2O2. Catalase activity was expressed in U per milligram of protein.

The activity of glutathione S-transferase (GST) was assessed and was carried out, according to the formation of 2,4-dinitrochlorobenzene with glutathione conjugate ( 21 ). To this end, 1 mmol/L of CDNB was added to the buffer containing 1 mmol/L of GSH and the aliquot (10 µL) of the plasma sample. After the addition of CDNB, the change was monitored in absorbance at 340 nm for 90s. The molar extinction coefficient used for CDNB was ∈340 = 9,6 mmol/L × cm. One unit of GST activity was defined as the amount of enzyme that catalyzed the formation of one mole of product /min/mL. GST activity was expressed in μmole/min/g.

Lipid peroxidation was assessed by malondialdehyde (MDA) concentrations, following the methodology prescribed by Buege and Aust ( 22 ) with modifications. An aliquot of 200 µL plasma was homogenized in 400 µL TBARS solution (15% trichloroacetic acid and 0.375% thiobarbituric acid dissolved in 0.25 N HCL1). The mixture was kept in a water bath at 90°C for 40 minutes. After this time, the mixture was cooled in an ice bath and 600 µL of N-butanol was added with subsequent vortexing for 1 minute and centrifugated for 10 minutes at 2500 rpm. An aliquot (200 μL) of the supernatant was used to measure absorbance at 535 nm. MDA concentration was determined using a standard curve of known concentrations of 1,1,3,3-tetramethoxypropane (TMPO) (10 mM) diluted 1: 500 (v/v). MDA concentrations were expressed as µmol / mL plasma.

Protein oxidation was evaluated by the quantification of protein carbonyl using the method described by Mekrungruangwong and cols. ( 23 ) with modifications. The plasma sample was diluted (1:10) in saline. A 200 µL aliquot of the diluted sample was homogenized with 800 µL of dinitrophenylhydrazone (DNPH), diluted in 2.5M HCl (microtube A). Another 200 µL of diluted sample was mixed in 800 µL of 2,5M HCl (microtube B). Reactions were incubated at room temperature, in a dark place, for 1 hour. One milliliter of 20% trichloroacetic acid (TCA) was added to the two microtubes and centrifuged at 10,000xg for 10 minutes at 4°C. The supernatant was discarded. Soon after, the precipitate was resuspended in 1 mL of 1:1 (v/v) ethyl acetate: ethanol and centrifuged again at 10.000 xg, for 10 minutes, at 4ºC. This last step was performed again and after discarding the supernatant, the precipitate was resuspended in 500 µL guanidine hydrochloride and carried through new centrifugation at 10.00 xg for 5 minutes, at 4ºC. It was performed the reading of the supernatant absorbance at 370 nm. The carbonylated protein was expressed in nmol/mL.

Nitric oxide production was indirectly determined by the total nitrite dosage of the samples, using Griess’s reagent ( 24 ). Initially, 50 µL of plasma was incubated with 100 µL Griess reagent (1% sulfanilamide, 0.1% N-(1Naphthyl) ethylenediamine, and 2.5% H3PO4) at room temperature for 10 min in the dark and then, the absorbance reading was performed at 570nm. The absorbance conversion to micromolar concentrations of NO were obtained from a standard sodium nitrite curve (0–125 μM) and expressed as NO µmol/mg protein concentrations.

Antioxidant capacity of the plasma was evaluated by the ferric reducing antioxidant power (FRAP) assay ( 25 ). FRAP reagent was prepared as required by mixing acetate buffer (0.3 M, pH 3.6), TPTZ solution (2,4,6-tripyridyl-s-triazine, 10 mM), and ferric chloride (FeCl3r6H2O) solution (0.02 M). The assembly of the standard curve was performed using a solution of ferrous sulfate in acetate buffer (0.0695 g ferrous sulfate (FeSO4.7H2O) in 5 mL of 0.3M acetate buffer). In an ELISA plate, a 10 µL aliquot of ultrapure water and 300 µL of FRAP solution were homogenized to make blank, 10 µL of each standard curve point added with 300 µL of FRAP solution and 10 µL of plasma added 300 µL of FRAP solution. Reactions were incubated at 37ºC for 4 minutes and the absorbance reading was taken at 595nm. Changes in absorbance in relation to white were recorded and FRAP was expressed in μmol/L.
